# The Effects of *Radix isatidis* Raw Material on Egg Quality, Serum Biochemistry, Gut Morphology and Gut Flora

**DOI:** 10.3390/antiox12122084

**Published:** 2023-12-07

**Authors:** Pingping Li, Zenghao Yan, Panpan Shi, Deqin Wang, Zhenhui Liu, Mengting Lu, Chuyuan Li, Yulong Yin, Peng Huang

**Affiliations:** 1 College of Animal Science and Technology, Hunan Agricultural University, Changsha 410128, China; 488162468@stu.hunau.edu.cn (P.L.); 1140110690@stu.hunau.edu.cn (P.S.); 18363366773@163.com (Z.L.); 15274384669@163.com (M.L.); 2 Hutchison Whampoa Guangzhou Baiyunshan Chinese Medicine Co., Ltd., Guangzhou 510515, China; yanzengh@mail3.sysu.edu.cn (Z.Y.); wdq2008@163.com (D.W.); 3 Hunan Key Laboratory of Traditional Chinese Veterinary Medicine, Hunan Agricultural University, Changsha 410128, China

**Keywords:** antioxidant status, anti-inflammatory, egg quality, RIHR, gut microbiota

## Abstract

China produces more than 30 million tons of drug residues every year. Therefore, innovative solutions are needed to mitigate environmental damage. Certain plant compounds boost hens’ health and performance. *Radix isatidis* is promising for layer production. This study elucidates the multidimensional impact of *Radix isatidis* residual material (RIHR) on laying hens, focusing on the egg quality, intestinal health and the microbial landscape. A total of 288 55-week-old Peking powder laying hens with similar laying rates and body weights were randomly divided into four groups, with eight replicates per group and nine hens per replicate. The groups were divided into a control group, an RIHR low-dose group, a medium-dose group and a high-dose group according to a single-factor, completely randomized design. For the three RIHR treatment groups, the added amounts were 5 kg/t, 10 kg/t and 15 kg/t, respectively. Liquid chromatography- mass spectrometry (LC-MS), molecular docking, fluorescence quantitative PCR and other methods were used. The results showed that three main anti-inflammatory and antiviral compounds were identified in RIHR-indirubin (0.21 μg/g), deoxyvasicinone (0.18 μg/g) and epigoitrin (0.39 μg/g). RIHR significantly increased the eggshell thickness, Haugh unit and protein height (*p* < 0.05). It also had significant antioxidant and anti-inflammatory effects on ilea and ceca (*p* < 0.05). The microbial analysis demonstrated that RIHR supplementation led to a significant reduction in foregut *Lactobacillus* levels (*p* < 0.05). In the hindgut, a significant increase in pathogenic bacteria was observed (*p* < 0.05). The study concludes that RIHR’s anti-inflammatory compounds may directly act on the intestinal tract to modulate inflammation, highlighting its potential for targeted interventions in poultry health and nutrition.

## 1. Introduction

Recent estimates reveal a concerning environmental impact from China’s traditional Chinese medicine (TCM) sector, with an annual accumulation of pharmaceutical residues exceeding 30 million tons [1]. While a fraction of these herbal medicinal remnants has been repurposed for endeavors such as edible mushroom cultivation, organic fertilizer production, animal feed and paper manufacturing, the resultant products exhibit a conspicuous dearth of added value, potentially exacerbating secondary environmental pollution [2,3]. Herbal tea residues (HTRs), often discarded post production, hold numerous nutrients and bioactive compounds. In one study, where these discarded HTRs underwent fermentation and were used as feed, they exhibited a stabilizing effect on the physiological functions of black goats [4]. Moreover, the research indicated that utilizing ginseng dregs as a raw material enabled the co-production of ginseng polysaccharides, ginsenosides and succinic acid, thus adding significant value to what was once considered waste [5]. These findings underscore the potential of reusing herbal dregs, integrating waste management with practical production.

In fact, the bioactive constituents of some botanicals augment the processes of digestion, absorption and immunity in laying hens while ameliorating intestinal inflammatory responses, thereby bolstering production performance [6]. For instance, *Ligustrum lucidum*, *Schisandra chinensis* and *Allium tuberosum* demonstrate antiviral and antibacterial attributes [7]. *Radix isatidis*, a ubiquitous botanical specimen in China, harbors a plethora of anti-inflammatory, antibacterial and antiviral properties [8,9]. Its principal constituents encompass Isatidis Radix polysaccharides, indoles, indirubin, sterols and steroids. The polysaccharides derived from *R. isatidis* exhibit heightened concentrations and pharmacological benefits akin to the herb itself [10]. The chemical composition of *R. isatidis* is highly complex, and nearly 200 compounds have been isolated from it, including alkaloids, lignans, organic acids and flavonoids [11]. Among these, the alkaloids have been the most studied and exhibit notable bioactivity [12]. Indirubin is the main bioactive component of the traditional Chinese medicine Indigo naturalis and is a bisindole alkaloid. Multiple studies have shown that indirubin exhibits good anticancer, anti-inflammatory and neuroprotective properties [13]. It has been reported that indirubin administered at doses of 25, 50 and 100 μM has anti-inflammatory effects on mice [14]. Epigoitrin is extracted from the root of *R. isatidis* and exhibits antiviral activity, inhibiting viral attachment and proliferation [15]. It was reported that 88 mg/kg of epigoitrin significantly decreased the susceptibility of restraint mice to influenza virus, as evidenced by a lowered mortality, attenuated inflammation and decreased viral replications in lungs [16]. Given its multifaceted nature, *R. isatidis* is acknowledged for its substantial potential in the domain of laying hen production. Serving as environmentally friendly, secure and cost-effective feed additives, the residues from *R. isatidis* will probably assume a pivotal role in the cultivation of laying hens.

Our research focused on the multidimensional impact of *R. isatidis* residual material on laying hens, specifically focusing on the interplay between egg quality, intestinal health and the gut microbiome. More importantly, using sophisticated techniques, including quantitative fluorescence validation and Liquid chromatography- mass spectrometry (LC-MS), the study identified three potent anti-inflammatory and antiviral compounds in RIHR and elucidated their important role in promoting gut health. This ground-breaking research uncovers the multiple benefits of *R. isatidis* residual material, revealing its remarkable potential to improve egg quality and reduce inflammation in laying hens. Consequently, the results provide a foundation for the practical application of RIHR in actual production.

## 2. Materials and Methods

### 2.1. Preparation of R. isatidis Raw Materials

To prepare the *R. isatidis* raw material, add water to the extract, boil slightly, set at 100 °C and extract the *R. isatidis* with water twice, the first extraction for 2 h and the second extraction for 1 h. Regarding the amount of solvent, add 10 parts of water the first time and add eight parts of water the second time. Then, after separating the supernatant, what remains is the *R. isatidis* residual material that was used for the feed additive. After its preparation, the remaining *R. isatidis* residual material was eventually ground into a dry powder and added to the feed for laying hens. The size of the powder was 30 mesh.

### 2.2. Determination of Routine Nutritional Components of RIHR

#### 2.2.1. Determination of Amino Acids by High Performance Liquid Chromatography (HPLC)

For the preparation of amino acid mixed standard series solutions, seventeen types of amino acid mixed standard solutions (each with a concentration of 1.0 mmol·L^−1^ for every amino acid) were diluted with 0.1 mol·L^−1^ diluted hydrochloric acid to create mixed standard series solutions with different concentration gradients. For sample treatment, the HVP powder was diluted 30 times with water and then diluted 5 times with 0.1 mol·L^−1^ hydrochloric acid. After diluting HVP and soy sauce 10 times with water, they were diluted 5 times with 0.1 mol·L^−1^ hydrochloric acid. To derivatize, absorb 100 μL of amino acid mixed labeling solution, HVP treatment solution, or diluted sauce release solution and transfer it into a 2 mL centrifuge tube. Add 100 μL of 0.1 mol·L^−1^ Na_2_B_4_O_7_ aqueous solution and 100 μL of 1% DNFB acetonitrile solution, then cover it tightly and shake well. The reaction was carried out in a constant-temperature water bath at 60 °C for 1 h. After the reaction, the test tube was cooled in cold water, and 0.02 mol·L^−1^ Na_2_HPO_4_ and 0.02 mol·L^−1^ NaH_2_PO_4_ aqueous solutions were added to a total volume of 1 mL, mixed, and filtered with a 0.45 μm organic membrane for measurement.

#### 2.2.2. Determination of Conventional Nutrients in Feed

The content of crude protein, crude fat, moisture, crude fiber, crude ash, calcium and total phosphorus in RIHR were determined by AOAC’s official method [17]. Energy determination was carried out as follows: Energy was determined by using oxygen bomb calorimeter. Initially, a weighed (1.0 g) feed sample was put into the oxygen bomb cylinder of the oxygen bomb calorimeter, and then, it was filled with oxygen at a certain pressure and electrified to burn. The heat released from the combustion of the feed sample was transferred through the wall of the bomb and absorbed by the constant-temperature water outside the bomb cylinder. The energy value of the feed sample can be calculated based on the difference in water temperature before and after the combustion.

### 2.3. Analysis of Chemical Constituents in the RIHR by LC-MS

By optimizing the chromatographic and mass spectrometric conditions, the final LC-MS detection conditions were established as follows: Chromatographic conditions: The chromatographic column was Agilent Eclipse XDB-C18 (2.1 mm × 150 mm, 1.8 μm). The mobile phase comprised 0.1% formic acid in the aqueous phase (A) and acetonitrile in the organic phase (B). The gradient elution procedure was as follows: 0–8 min, 2–30% B; 8–25 min, 30–95% B; 25–30 min, 95% B; and 30.1–35 min, 2% B. The column temperature was set at 35 °C. The autosampler temperature was maintained at room temperature (25 ± 2 °C). A flow rate of 0.3 mL/min was used with a sample size of 1 μL. The DAD collected data via full wavelength scanning within the range of 210–400 nm.

### 2.4. Experimental Animals and Experimental Design

The study was approved by the Animal Ethical Committee of Hunan Agricultural University. A total of 288 55-week-old Peking powder laying hens with similar laying rate and body weight were randomly divided into four groups, with eight replicates per group and nine hens per replicate. The groups were divided into a control group, an RIHR low-dose group, a medium-dose group and a high-dose group according to a single-factor, completely randomized design. The control group received a basal diet, while the low-, medium- and high-dose groups were fed experimental diets supplemented with RIHR. The supplementation levels were 5 kg/t, 10 kg/t and 15 kg/t, respectively. During the prefeeding period of the first week, all chickens were fed the basal diet. At the end of the prefeeding period, the production performance of each group was measured, so that there was no significant difference in laying rate among the groups, and the formal experiment was started. The experiment lasted for 63 days, during which the laying rate, egg weights, and feed intake of laying hens were recorded.

### 2.5. Feeding Management and Index Determination

#### 2.5.1. Laying Hen Diet and Management

According to China’s Chicken Feeding Standard (NY/T33-2004) [18], the dietary formula to meet the nutritional needs of laying hens during the peak laying period was formulated. The dietary ingredients and nutrient composition are shown in Table 1.

#### 2.5.2. Determination of Production Performance of Laying Hens

The experiment lasted for 63 days. Laying rate, average daily feed intake and feed-to-egg ratio of laying hens were recorded in the first and eighth weeks. The production performance is calculated as follows:

1. $Laying\;rate=\frac{Number\;of\;eggs\;laid}{Number\;of\;hens\;on\;the\;day}$ × 100%
2. $Average\;daily\;feed\;intake=\frac{Total\;Feed\;Intake}{Test\;Days\times Number\;ofchickens\;tested}$
3. $Feed\;to\;egg\;ratio=\frac{average\;daily\;feed\;intake}{daily\;egg\;weight}$
4. Egg weight: Measured with Egg Analyzer, accurate to 0.01 g.

#### 2.5.3. Determination of Egg Quality

Egg samples were collected on the day before the end of the experiment. Initially, at the beginning of the experiment, the egg characteristics within each group were assessed to ensure a relatively consistent egg size, aiming for medium-sized eggs to reduce experimental error. Eight chickens were selected from each group, and three replicates of eggs from each chicken were selected for determining egg quality. Therefore, there were twenty-four eggs from each group for assessing egg quality. The following parameters were measured:

1. Egg shape index: The transverse and longitudinal diameters of each egg were determined using electronic digital vernier calipers and then calculated.
  $$Egg\;shape\;index=\frac{Transverse\;Diameter}{Longitudinal\;Diameter}\times 100\%$$
2. Eggshell strength: The intact eggs were tested on a THV-1DX dual indenter micro- Vickers hardness tester.
3. Yolk color: The egg is broken and tested in the Egg Analyzer.
4. Protein height and Haugh unit: The Egg is broken and tested in the Egg Analyzer.
5. Eggshell thickness: The eggshell thickness meter is utilized to measure the eggshell thickness of the sharp end, blunt end and the middle part of the egg, with the average value being considered the overall eggshell thickness.
6. Yolk index: Two egg yolks were selected for each replicate, and their height and diameter were measured using an electronic digital vernier caliper.
  $$Yolk\;index=\frac{Yolk\;Height}{Yolk\;Diameter}$$
7. Yolk ratio: The proteinaceous material covering the yolk surface, along with any adhering remnants, was eliminated, following which the yolk weight was recorded using an analytical balance.
  $$Yolk\;ratio=\frac{Yolk\;Weight}{Whole\;Egg\;Weight}$$

#### 2.5.4. Determination of Serum Biochemical Indexes

The serum levels of alkaline phosphatase (ALP), aspartate aminotransferase (AST), alanine aminotransferase (ALT), blood phosphorus (IP), calcium (Ca), albumin (ALB), glucose (GLU), total cholesterol (TC), triglyceride (TG), high-density lipoprotein (HDLC) and low-density lipoprotein (LDLC) were measured in laying hens using a Kehua automatic biochemical analyzer. Additionally, the levels of immunoglobulin A (IgA), immunoglobulin G (IgG) and immunoglobulin M (IgM) were also assessed. Eight samples were selected for detection in each group, and the serum biochemical kits were purchased from Kehua Bioengineering Co, Ltd., Shanghai, China.

#### 2.5.5. Determination of Antioxidant Index

On the day before the end of the experiment, 8 eggs were randomly selected from each group. The yolks of the selected eggs were separated from the white and stored at −30 °C. The content of total antioxidant capacity (T-AOC) in egg yolks and malondialdehyde (MDA) and superoxide dismutase (SOD) in liver were determined according to the instructions of the kit. All of the kits were purchased from Boxangong Technology Co., Ltd., Beijing, China.

#### 2.5.6. Determination of Serum Immune Indexes

Immunoglobulin A (IgA), Immunoglobulin G (IgG) and D-Lactic acid were measured in the control group and the optimal group by enzyme-linked immunoassay. Eight samples were selected from each group for testing. All of the kits were purchased from Jiancheng Biological Co., Ltd., Nanjing, China.

#### 2.5.7. Histological Analysis

After 4% paraformaldehyde-fixed intestinal samples of laying hens were prepared into paraffin sections, Villus height and Crypt depth of duodenum, jejunum and ileum were measured using image analysis software, and villus height/crypt depth (V/C) was calculated.

#### 2.5.8. Fluorescent Quantitative PCR

The levels of ileum- and cecum inflammation-related genes (*IL-1β*, *IL-6*, *IL-4*, *IL-10*, *NF-KB*, *COX2*, *TNF-α*), antioxidant genes (*NQO1*), and intestinal barrier genes (*Occludin*) in the control group and the optimal group were detected by qPCR. β-actin was used as a reference gene for normalization. Additional information about the primers used in this study is provided in Table S1. All primers were designed using chicken sequences from Gene Bank and synthesized by a biotechnology company (Sagon Biotech, Shanghai, China). The total RNA of cecum tissue samples was extracted with Trizol according to the manufacturer’s instructions (Solarbio, Beijing, China), and the integrity of the RNA was assessed by visualization on agarose gel. RNA concentration and purity were determined using a Nanodrop ND-2000 spectrophotometer (Thermo Scientific, Ottawa, ON, Canada). cDNA synthesis was carried out according to the instructions of the kit manufacturer (Takara Biotechnology, Dalian, China). Quantitative real-time PCR was performed on a CFX Connect Real-Time PCR Detection System (ANALYTIKJENA, Jena, Germany.) using a SYBR Green PCR kit (Takara Biotechnology, Dalian, China). Results were calculated using the 22DDCT method (Livak K.J. and Schmittgen, T.D. 2001).

#### 2.5.9. Molecular Docking Simulation

First, the crystal structure of the gene was downloaded from the RCSB Protein Data Bank30 (https://www.rcsb.org/ accessed on 5 June 2023.), and the small molecule was downloaded from TCMSP MENU. Then, molecular docking software (https://www.dockeasy.cn/DockCompound# accessed on 5 June 2023) was used for molecular docking. Finally, PyMol2.3.0 software was used to realize the visualization of the binding model.

### 2.6. Gut Microbiota Analysis

For this analysis, 16S rDNA high-throughput sequencing technology was utilized to analyze the microbial composition of the anterior and posterior intestinal contents. Initially, Illumina sequencing generated PE reads that were merged based on the overlap relationship, followed by quality control and filtering of the sequence data. Then, the samples were classified into operational taxonomic units (OTUs) and identified taxonomically. Various diversity indices were analyzed based on the OTUs. Additionally, OTUs were analyzed for diversity indices and sequence depth. Statistical analyses of community structure were performed at various classification levels based on taxonomic information. Based on the above analyses, further statistical and visualization analyses, such as multivariate analysis and significance testing for variations in community composition and phylogenetic information, were conducted for multiple samples.

### 2.7. Statistical Analysis

The test data were organized using Excel 2019 software. The performance, egg quality, and serum biochemical data were analyzed using the Tukey–Kramer method in SPSS23.0 statistical software. The section data were analyzed by one-way analysis of variance. Based on the above data, an independent sample *t*-test analysis was conducted for RIHR-H in subsequent experiments. All results were represented by “mean ± standard error”, with mean being 8 repetitions. *p* < 0.05 was considered significant difference, *p* < 0.01 was considered extremely significant difference. All data were mapped using GraphPad Prism 9.0 software, with * representing *p* < 0.05 and ** representing *p* < 0.01.

## 3. Results

### 3.1. Determination of Conventional Nutritional Components of RIHR

The determination results of amino acids in RIHR are shown in Table S2, with a total of seventeen identified amino acids: ASP, GLU, SER, HIS, GLY, THR, ARG, ALA, TYR, VAL, MET, ILE, LEU, PHE, LYS, PRO and CYS. The respective content of each amino acid is 0.59%, 0.8%, 0.28%, 0.22%, 0.34%, 0.3%, 0.94%, 0.33%, 0.15%, 0.39%, 0.05%, 0.31%, 0.28%, 0.46%, 0.37%, 0.73% and 0.07%.

The analysis results of the conventional nutritional components of RIHR are presented in Table S3. The identified contents of crude protein, energy, moisture, coarse ash, crude fat, coarse fiber, calcium and phosphorus are 12.13%, 5147.08 cal/g, 6.65%, 7.17%, 7.5%, 8.95%, 1.18% and 1.51%, respectively.

### 3.2. Qualitative Results for Main Chemical Constituents in RIHR

The mass spectrometry analysis identified a total of eight chemical components in RIHR, including L-arginine (1.19 μg/g), guanine (1.02 μg/g), L-phenylalanine (0.19 μg/g), Epigoitrin (0.39 μg/g), deoxyvasicinone (0.18 μg/g), 3-indole acetonitrile (0.52 μg/g), indigo (1.90 μg/g), and indirubin (0.21 μg/g) (Table 2). The three active ingredients, indirubin, deoxyvasicinone and epigoitrin, are of scientific value. Indirubin is the main bioactive component of the traditional Chinese medicine Indigo naturalis and is a bisindole alkaloid. Indirubin exhibits good anticancer and anti-inflammatory activity. Deoxyvasicinone is an effective component of medicinal plants such as *Adhatoda Vasica Nees* and *Peganum harmala L*, which exhibit anti-inflammatory, antioxidant, antibacterial and other biological activities with a low toxicity and high safety. Epigoitrin is extracted from the root of *R. isatidis* and exhibits antiviral activity by inhibiting viral attachment and proliferation. Therefore, these three bioactive compounds highlight the potential of RIHR to treat inflammation.

### 3.3. Effect of RIHR on Production Performance of Laying Hens

The effects of RIHR on the production performance of laying hens are shown in Table 3. In the first week, compared with the control group, the laying rate in the RIHR-L group was increased (*p* > 0.05), the average daily feed intake was significantly decreased (*p* < 0.05), the feed-to-egg ratio was significantly decreased (*p* < 0.05), and the egg weight increased in the RIHR-L and RIHR-M groups, but the difference was not significant (*p* > 0.05). In the eighth week, compared with the control group, the laying rate decreased in each RIHR dose group, the average daily feed intake decreased in the RIHR-H group, and the feed–egg ratio decreased in the RIHR-M group, although these differences were not significant (*p* > 0.05). However, the egg weight significantly increased in the low-, medium- and high-dose RIHR groups (*p* < 0.05).

### 3.4. Effect of RIHR on Egg Quality

The effect of RIHR on the egg quality showed that the egg shape index, yolk color, Haugh unit, protein height and eggshell thickness in the low-, medium- and high-dose RIHR groups significantly increased compared with the control group (*p* < 0.05). The other indexes had no significant effect (*p* > 0.05) (Table 4).

### 3.5. Effects of RIHR on Serum Biochemical Indices of Laying Hens

The effects of RIHR on the serum biochemistry of laying hens are shown in Table 5. Compared with the control group, the contents of lg M, lg A, IP and Ca in the RIHR medium- and high-dose groups were increased, but the differences were not significant (*p* > 0.05). The contents of ALT and AST in the high-dose group were decreased, but the differences were not significant (*p* > 0.05). HDL-C significantly increased in the medium- and high-dose groups (*p* < 0.05).

### 3.6. Effects of RIHR on Antioxidant Capacity and Immunity of Laying Hens

**Figure 1:**
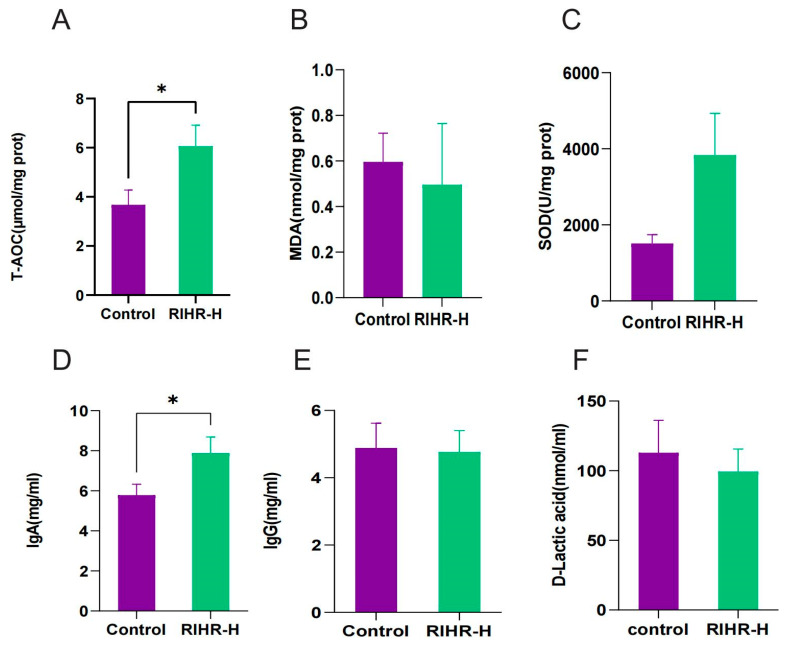
Effects of RIHR on antioxidant capacity and immunity of laying hens. (**A**) Effect of high dose of RIHR on T-AOC in egg yolk of laying hens. (**B**) Effect of high dose of RIHR on liver MDA of laying hens. (**C**) Effect of high dose of RIHR on SOD in liver of laying hens. (**D**–**F**) Effects of RIHR on serum immune indexes. * In the figure indicates a significant difference (*p* < 0,05), while no * indicates no significant difference (*p* > 0.05).

In order to further explore the effects of RIHR on the antioxidant capacity and immunity of laying hens, antioxidant indices in egg yolk and liver, as well as immunological indices in serum, were determined in the optimal-dose group (RIHR-H). The results are shown in Figure 1A–C, and compared with the control group, the RIHR-H group significantly increased the total antioxidant capacity (T-AOC) of the yolk (*p* < 0.05), while it decreased the level of MDA in the liver (*p* > 0.05) and increased the capacity for superoxide dismutase (SOD) in the liver (*p* > 0.05). This indicates that RIHR can improve the antioxidant capacity of egg yolks and the liver in laying hens, promoting their health and improving egg quality. 

The determination of serum immune indexes is shown in Figure 1D–F. Compared with the control group, the RIHR-H group significantly increased the content of IgA (*p* < 0.05) and decreased the content of D-lactic acid (*p* > 0.05), but it showed no significant difference in IgG (*p* > 0.05). This suggests that RIHR can also affect and enhance the immunity of laying hens.

### 3.7. Effects of RIHR on the Intestinal Histomorphology of Laying Hens

**Figure 2:**
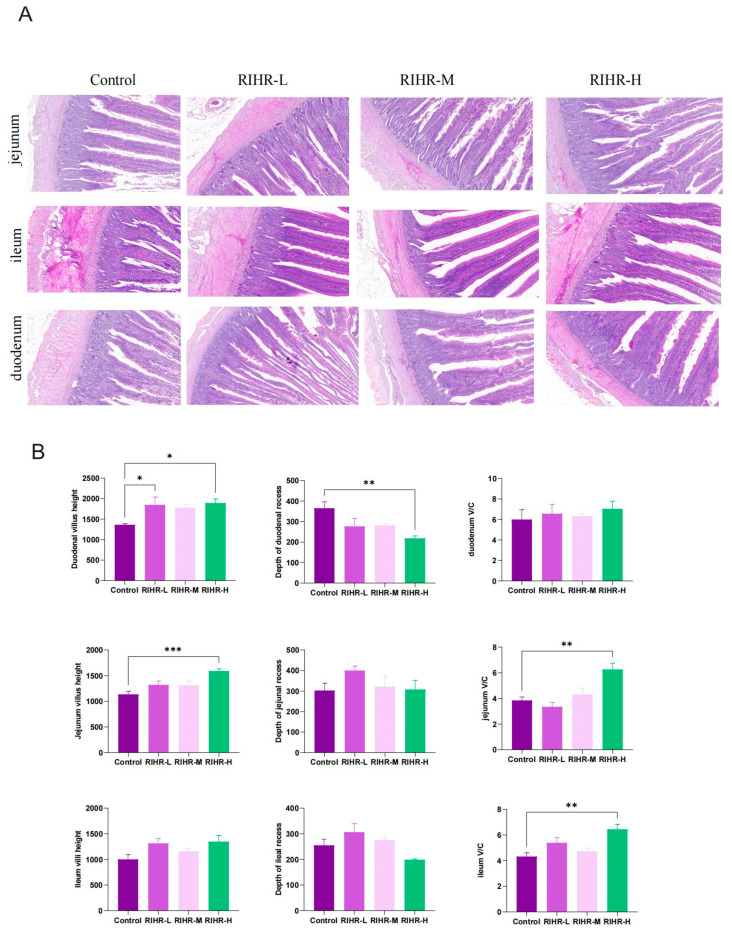
Effect of RIHR on intestinal tract of laying hens. (**A**) Effect of RIHR on intestinal section of laying hens. The slice multiple is 10×. (**B**) Effects of RIHR on intestinal morphology of laying hens. * Indicates significant difference (*p* < 0.05), ** and *** indicate extremely significant differences (*p* < 0.01), and no * indicates no significant difference (*p* > 0.05).

The impact of RIHR on the intestinal morphology of laying hens is depicted in Figure 2A. The duodenal segments exhibited elongated intestinal villi in the medium-dose group, characterized by an orderly arrangement and well-defined brush borders. Meanwhile, the high-dose group exhibited the most distinct brush borders, an increased abundance of goblet cells, a homogeneous distribution pattern and a moderate depth of crypts. The number of goblet cells in the control, low-dose, and medium-dose groups was comparatively lower than that in the high-dose group. Additionally, the experimental groups exhibited an enhanced villus length and reduced crypt depth when compared with the control group. The low-to-moderate doses of RIHR resulted in an increased height and width of the villi, along with closely aligned columnar epithelial cells. Observations of the jejunum revealed that the villous epithelial cells in the control group were well-formed and clearly defined, although with fewer goblet cells and a more complete population of crypt cells. Conversely, the medium-dose group exhibited thinner villi in the jejunum, a higher presence of goblet cells and a more comprehensive morphology, structure and crypt count. In the high-dose group, the villi in the small intestine appeared indistinct, accompanied by a reduced number of goblet cells. 

The effects of RIHR on the intestinal villus height and intestinal ratio of laying hens are shown in Figure 2B. Compared with the control group, the duodenal villus height (*p* < 0.05), jejunal villus height (*p* < 0.01), jejunal ratio (*p* < 0.01) and ileum ratio (*p* < 0.01) were significantly increased in the RIHR high-dose group. The depth of the duodenal recess decreased (*p* < 0.01).

### 3.8. Effects of RIHR on the Expression of Related Genes in the Ileum and Cecum of Laying Hens

**Figure 3:**
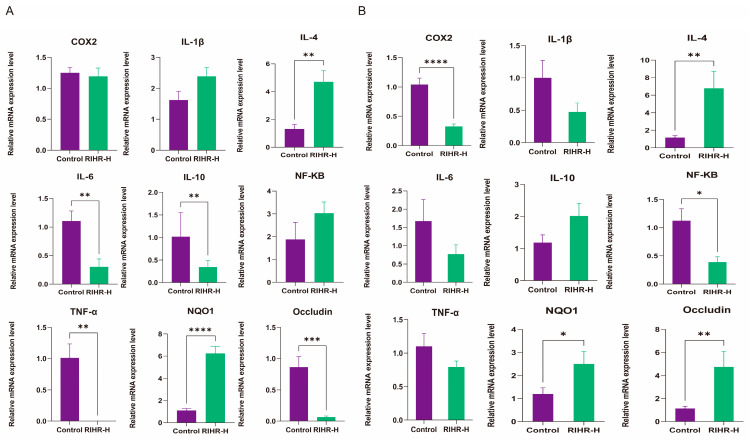
Effect of RIHR on expression of intestinal-related genes. (**A**) Effect of RIHR on expression of related genes in ileum. (**B**) Effects of RIHR on expression of cecum-related genes of laying hens. * Indicates significant difference (*p* < 0.05), **, *** and **** indicate extremely significant differences (*p* < 0.01), and no * indicates no significant difference (*p* > 0.05).

The effect of RIHR on the expression of related genes in the ileum is shown in Figure 3A. Compared with the control group, the levels of inflammatory factors *IL−6* (*p* < 0.01), *IL−10* (*p* < 0.01), *TNF−α* (*p* < 0.01), and *Occludin* (*p* < 0.01) were significantly decreased in the high-dose group of the RIHR, and the levels of the anti-inflammatory factor *IL−4* (*p* < 0.01) and the antioxidant factor *NQO1* (*p* < 0.01) were significantly increased. There were no significant effects of other indicators (*p* > 0.05).

The effects of RIHR on the cecum-related gene expression of laying hens are shown in Figure 3B. Compared with the control group, the expression levels of inflammatory factors *NF−KB* (*p* < 0.05) and *COX2* (*p* < 0.01) were significantly decreased in the RIHR high-dose group, and the expression levels of anti-inflammatory factor *IL−4* (*p* < 0.01) were also significantly increased. The high dose of RIHR significantly increased the expression of intestinal barrier *Occludin* (*p* < 0.01) and decreased the levels of inflammatory factors *IL−1β*, *TNF−α* and *IL−6*, but the difference was not significant (*p* > 0.05). Additionally, it also increased the levels of the anti-inflammatory factor *IL-10* (*p* > 0.05) and the antioxidant factor *NQO1* (*p* < 0.05).

### 3.9. RIHR Docked with Related Protein Molecules

**Figure 4:**
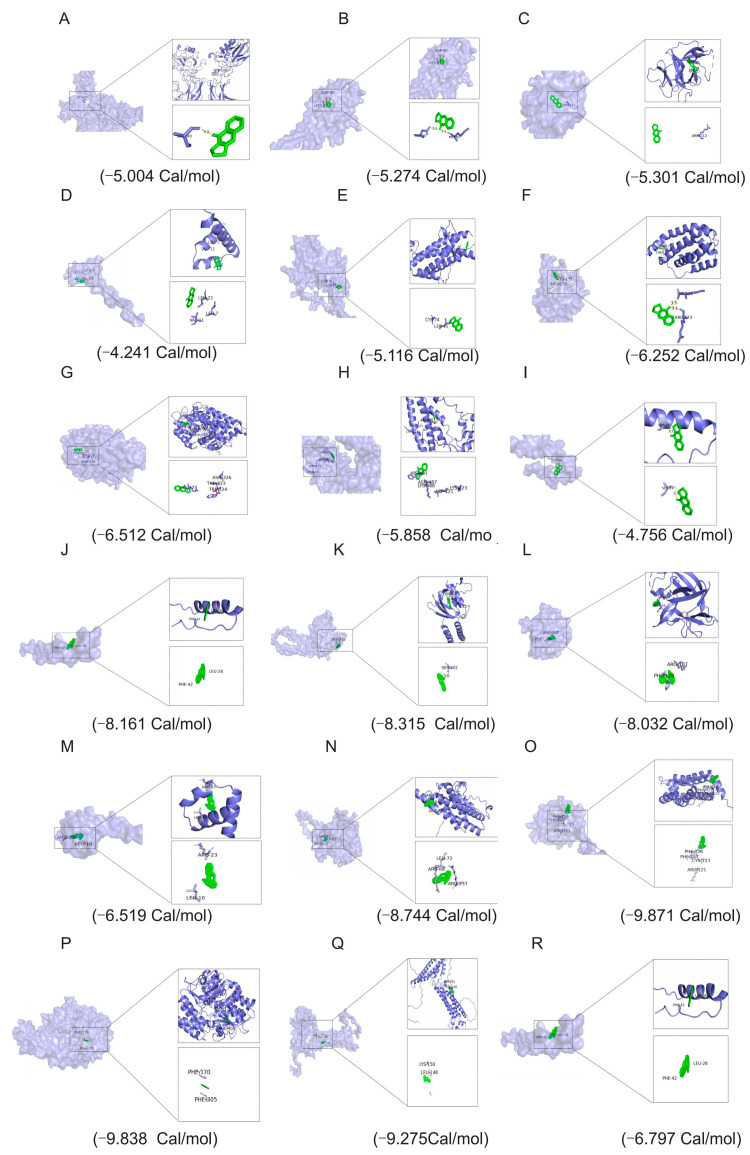
Molecular docking. (**A**) Deoxyvasicinone is docked with NF−KB molecule. (**B**) Deoxyva sicinone is docked with COX2 molecule. (**C**) Deoxyvasicinone is docked with IL−1β molecule. (**D**) Deoxyvasicinone is docked with IL−4 molecule. (**E**) Deoxyvasicinone is docked with IL−6 molecule. (**F**) Deoxyvasicinone is docked with IL−10 molecule. (**G**) Deoxyvasicinone is docked with NQO1 molecule. (**H**) Deoxyvasicinone is docked with Occludin molecule. (**I**) Deoxyvasicinone is docked with TNF−α molecule. (**J**–**R**) Indirubin molecular docking.

The molecular docking results showed that the binding energy of deoxyvasicinone to NF−KB was −5.004 Cal/mol, and the ligand formed a hydrogen bond with residues of SER-240. The hydrogen bond interaction distance was 3.3 A (Figure 4A). The binding energy of deoxyvasicinone to COX2 was −5.274 Cal/mol, and the ligand formed two hydrogen bonds with residues ASP-90 and MET-85. The hydrogen bond interaction distances were 3.1 A and 3.2 A (Figure 4B). The binding energy of deoxyvasicinone to IL−1β was −5.301 Cal/mol, and the ligand formed a hydrogen bond with the residue ARG-112 (Figure 4C). The binding energy of deoxyvasicinone to IL−4 was −4.241 Cal/mol, and the ligand formed a hydrogen bond with the residue LEU-22, LEU-7 and VAL-11 (Figure 4D). The binding energy of deoxyvasicinone to IL−6 was −5.116 Cal/mol, and the ligand formed a hydrogen bond with the residue CYS-74 and LEU-71 (Figure 4E). The binding energy of deoxyvasicinone to IL−10 was −6.252 Cal/mol, and the ligand formed a hydrogen bond with the residue LYS-175 and ARG-173, and the hydrogen bond interaction distances were 3.1 A and 3.5 A (Figure 4F). The binding energy of deoxyvasicinone to NQO1 was −6.512 Cal/mol, and the ligand formed a hydrogen bond with the residue LYS-175, ASN-326, THR-323 and TRP-324 (Figure 4G). The binding energy of deoxyvasicinone to Occludin was −5.858 Cal/mol, and the ligand formed a hydrogen bond with the residue HIS-491, ASN-487, LYS-488, ASP-421 and LYS-23 (Figure 4H). The binding energy of TNF−α was −4.756 Cal/mol, and the ligand formed four hydrogen bonds with residues SER-24. The hydrogen bond interactions were 3.3 A (Figure 4I).

The molecular docking results showed that the binding energy of indirubin and NF−KB was −8.161 Cal/mol, and the amino acids were PHE-298 and LYS-334. The binding energy of IL−6 was −8.744 Cal/mol, and the amino acids were LEU-72, ARG-68 and ARG-237. The binding energy of IL−10 was −9.871 Cal/mol, and the amino acids were PHE-126, PHE-127, CYS-123 and ARG-121. The binding energy of IL−4 was −6.519 Cal/mol, and the amino acids were ARG-23 and LEU-10. The binding energy of COX2 was −8.315 Cal/mol, and the amino acid was SER-222. The binding energy of TNF−α was −6.797 Cal/mol, and the amino acids were PHE-42 and LEU-28. The binding energy of IL−1β was −8.032 Cal/mol, and the amino acids were ARG-107 and PHE-128. The binding energy of NQO1 was −9.838 Cal/mol, and the amino acids were PHE-170 and PHE-305. The binding energy of Occludin was −9.275 Cal/mol, and the amino acids were LYS-150 and LEU-148 (Figure 4J–R).

The molecular docking results showed that the binding energy of Epigoitrin with NF−KB was −4.1 Cal/mol, and the amino acids were ASN-155 and ASP-151. The binding energy of IL−6 was −3.7 Cal/mol, and the amino acids were PHE-222, LEU-136 and LEU-71. The binding energy of IL−10 was −4.3 Cal/mol. The amino acids were LEU-33 and ILE-44. The binding energy of IL−4 was −3.0 Cal/mol, and the amino acids were LEU-10 and GLY-15. The binding energy of COX2 was −3.6 Cal/mol, and the amino acids were ILE-184, MET-86 and ASP-87. The binding energy of TNF−α was −3.2 Cal/mol, and the amino acids were CYS-43, PHE-42 and ILU-40. The binding energy of IL−1β was −3.5 Cal/mol. The amino acids were IEU-48, ALA-68 and PHE-110. The binding energy of NQO1 was −3.9 Cal/mol, and the amino acids were ARG-135, ASP-194 and ALA-193. The binding energy of Occludin was −3.6 Cal/mol. The amino acids were TYR-181, ALA-228 and ALA-72 (Figure S1).

### 3.10. Effects of RIHR on Microorganisms in the Foregut of Laying Hens

#### 3.10.1. Venn Diagram Analysis

**Figure 5:**
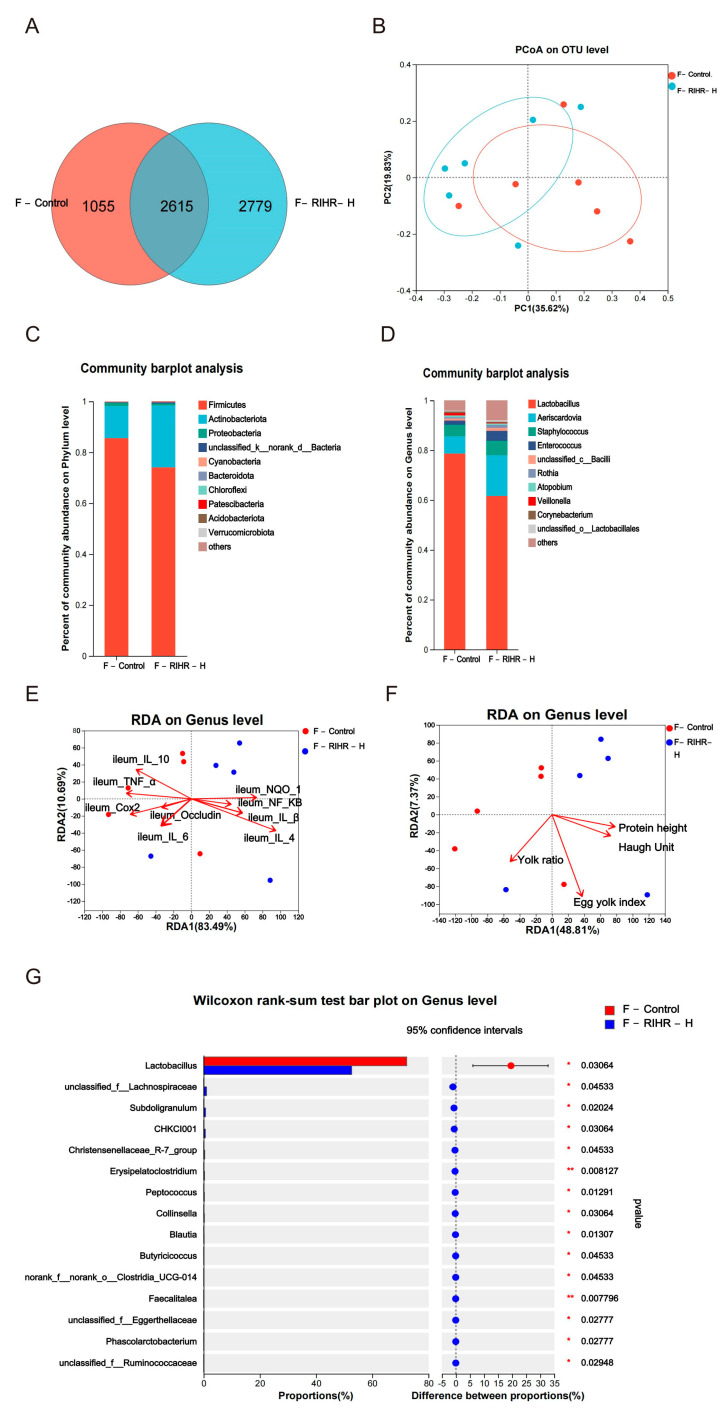
Effects of RIHR on microorganisms in the foregut of laying hens. (**A**) Venn analysis. (**B**) PCoA analysis. (**C**) Community heatmap analysis on Phylum level. (**D**) Community heatmap analysis on genus level. (**E**) RDA environmental factor correlation analysis. (**F**) RDA egg quality correlation analysis. (**G**) Difference test between control group and foregut of RIHR high-dose group (F-RIHR-H). * Indicates significant difference (*p* < 0.05), ** indicates extremely significant difference (*p* < 0.01).

The Venn diagram shows the unique and shared intestinal OTUs of different populations in foregut digesta. There were 2615 species shared between the F-Control group and F-RIHR-H. Specifically, there were 1055 species found in the specific environmental samples of the control group and 2779 species identified in specific environmental samples of the RIHR high-dose group (Figure 5A).

#### 3.10.2. Principal Component Analysis

To determine the potential effects of high doses of RIHR on the foregut microbiome of laying hens, PCoA (Principal Component Analysis) was performed to observe the differences in microbial characteristics between the two groups. The PCoA results showed a classification of the microbiome composition between the F-Control and F-RIHR-H groups. However, the two groups could not be clearly distinguished (Figure 5B).

#### 3.10.3. Diversity Analysis

Alpha diversity refers to the diversity of a particular region or ecosystem. In this functional module, species diversity is obtained by observing various index values, and statistical T-test is used to detect whether there are significant differences in index values between groups. Alpha diversity analysis was conducted based on the OTU. Compared with the F-Control, the Faith-pd index in the F-RIHR-H group exhibited a significant increase (*p* < 0.05), while the other indexes did not show significant differences (*p* < 0.05) (Figure S2).

The species richness at different taxonomic levels was analyzed to understand the composition of the communities studied. Histograms are used to visualize this information. At the phylum level, Firmicutes was the dominant group of all groups, followed by Actinobacteriota and Proteobacteria (Figure 5C). At the genus level, the dominant bacteria included *Lactobacillus*, *Aeriscardovia*, *Staphylococcus* and *Enterococcus*. The abundance of *Lactobacillus* decreased, while the abundance of *Aeriscardovia* and *Enterococcus* increased in the F-RIHR-H group compared with the F-Control group (Figure 5D). The RDA is used to reflect the relationship between flora and environmental factors. Therefore, RDA analyses were performed on the horizontal flora and ileal inflammatory factors and antioxidant-expressed genes at the genus level of the foregut of laying hens. The RDA results showed that the genus-level flora of the F-RIHR-H group was negatively correlated with the inflammatory factors *IL−6*, *TNF−α* and *COX2* and positively correlated with the antioxidant factor *NQO1* and the anti-inflammatory factor *IL−4*, when compared with the F-Control group (Figure 5E). On the other hand, RDA analyses were also carried out for indicators related to the foregut genus-level flora and egg quality in laying hens. The results showed a lower correlation with egg quality in the F-RIHR-H group compared with the F-Control group (Figure 5F). The abundance difference between the two groups at the genus level was analyzed by comparing the species abundance differences in the samples. The results showed that the abundance of *Lactobacillus* in the F-RIHR-H group was significantly decreased compared with the F-Control group (*p* < 0.05) (Figure 5G). 

**Figure 6:**
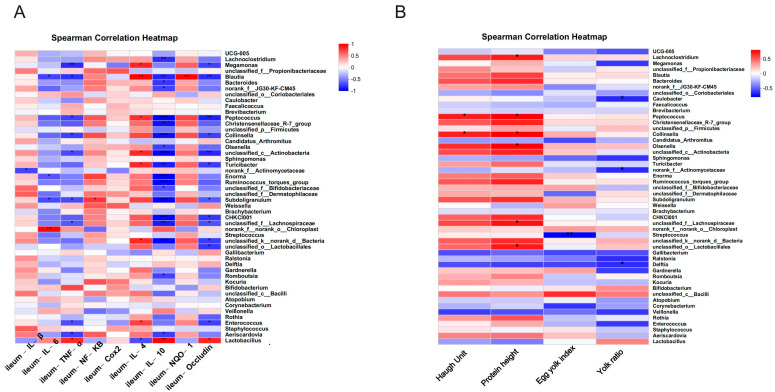
Correlation heatmap analysis. (**A**) Correlation heatmap of ileum expressed genes. (**B**) Heatmap of egg quality correlation. * Indicates significant difference (*p* < 0.05), ** and *** indicate extremely significant differences (*p* < 0.01).

**Figure 7:**
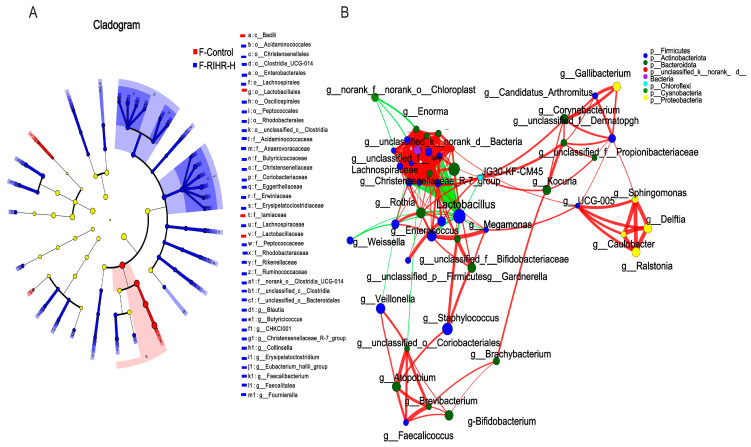
Comparative analysis of intestinal flora in RIHR. (**A**) Lefse analysis. (**B**) Network graph analysis.

An analysis was conducted to investigate the potential correlation between gut microbiota at the genus level and the expression levels of specific genes, including *IL−1β*, *IL−6*, *TNF−α*, *NF−KB*, *Cox2*, *IL−4*, *IL−10*, *NQO1* and *Occludin* in the ileum. This analysis aimed to gain a deeper understanding of the protective effects of RIHR on gut microbiota and parameters related to inflammation and antioxidation in laying hens. The results of the above genus level analysis suggest that RIHR treatment may affect the relative abundance of *Lactobacillus* in the foregut contents. Therefore, the correlation between *Lactobacillus* and specific genes was analyzed. A correlation heat map analysis showed that *Lactobacillus* was significantly positive correlated with *IL−10* (*p* < 0.01) and the intestinal barrier gene *Occludin* (*p* < 0.05), and *Enterococcus* had a significantly negative correlation with *TNF−α* (*p* < 0.05) (Figure 6A). Conversely, the potential correlation between genus-level intestinal flora and indicators related to egg quality, such as the Haugh unit, protein height, yolk index and yolk ratio, was also analyzed. This analysis was conducted to gain a deeper understanding of the effect of RIHR on egg quality improvement. The results of the heat map analysis of the correlation between intestinal flora and egg quality showed that *Collinsella* had a significant positive correlation with the Haugh unit and protein height (*p* < 0.05), while *Lactobacillus* had a negative correlation with the Haugh unit and protein height (*p* > 0.05) (Figure 6B). This suggests that the decrease in *Lactobacillus* does not impact egg quality, and the improvement in egg quality may be related to *Collinsella*. Linear discriminant analysis effect size (LEfSe) analysis is a method of discovering and interpreting markers of high-latitude data to identify features and their effects that best explain differences between species. The LEfSe taxonomic cladogram shows the key bacterial alterations, with different colors representing the different groups, and sizes of circles indicating the relative abundance. The results reveal significant variations in the composition of gut microbiota among the different groups. In particular, the LEfSe analysis identified a number of genera that act as biomarkers for taxa with notable differences among the two groups. In the foregut contents, there were thirty-five genera that were identified as biomarkers, with *g_Faecalitalea*, *f_Ruminococcaceae and g_Collinsella* being specific to the F-RIHR-H group (Figure 7A). 

The analysis of the network maps depicting the gut flora showed higher correlations among bacteria with higher abundances. In the whole network relationship, the number of positive correlations is significantly greater than the number of negative correlations. *Lactobacillus* were negatively correlated with most bacteria (Figure 7B). This suggests that RIHR may increase the number of other bacteria by reducing the number of *Lactobacillus*. 

### 3.11. Effects of RIHR on Posterior Intestinal Microorganisms of Laying Hens

#### 3.11.1. Venn Diagram Analysis

**Figure 8:**
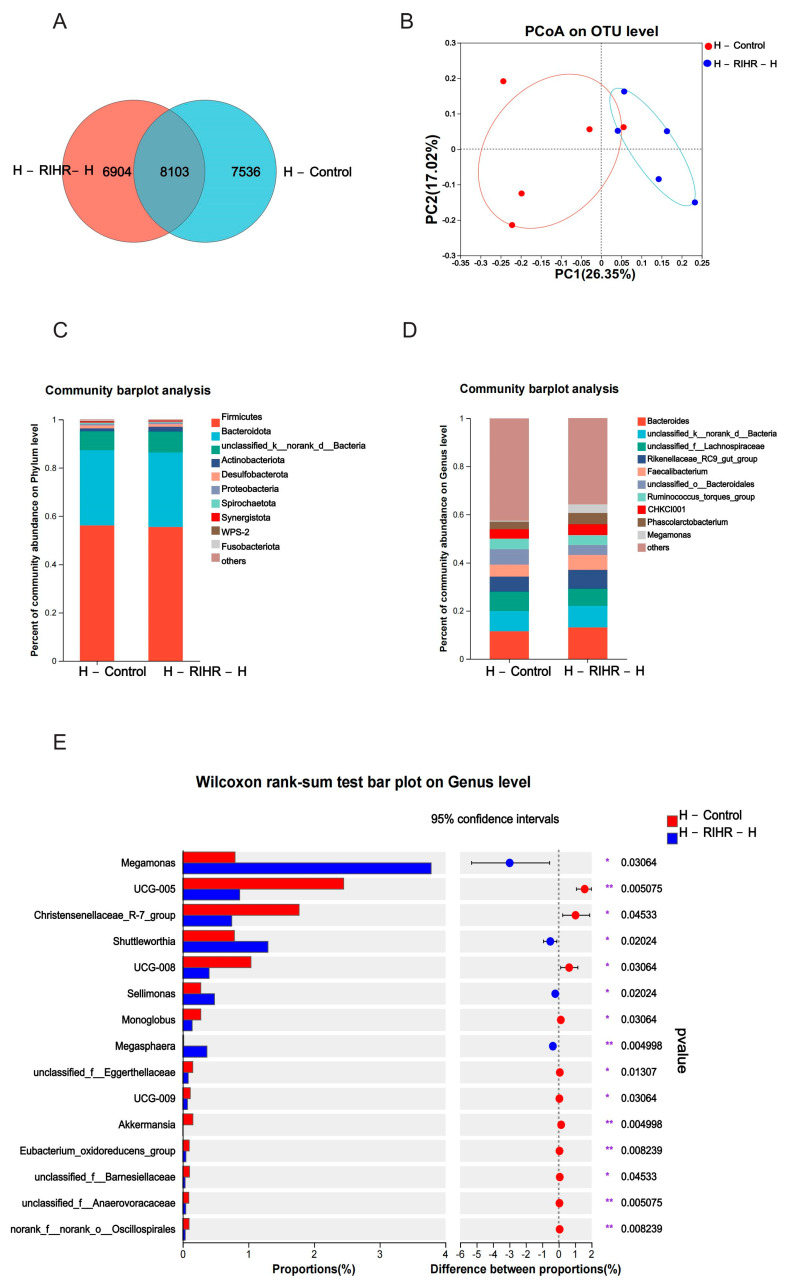
Effect of RIHR on posterior intestinal microorganisms of laying hens. (**A**) Venn analysis. (**B**) PCoA analysis. (**C**) Community heatmap analysis on Phylum level. (**D**) Community heatmap analysis on genus level. (**E**) Difference test between control group and foregut RIHR high-dose group (H-RIHR-H). * Indicates significant difference (*p* < 0.05), ** indicates extremely significant difference (*p* < 0.01).

Venn diagrams were used to assess the prevalence of common and exclusive species in multiple samples to gain an insight into the similarity of species composition in different environments in the sample. Each set of environmental samples has its own unique species, although these constitute a relatively small percentage of the overall species diversity. The overlap analysis of the species composition between the two groups showed that the number of species in the two groups was 8103. There were 7536 species in specific environmental samples of the control group and 6904 species in specific environmental samples of the RIHR high-dose group (Figure 8A).

#### 3.11.2. Principal Coordinates Analysis

High-throughput gene sequencing of 16SrRNA from hindgut DNA of laying hens was performed, and the sequencing data adequately reflected the richness and homogeneity of the microbial community in each sample. PCoA was used to distinguish the microbial composition of the sample group in the microbial scoring process. The results are shown in Figure 8B, and the control group and drug group were obviously separated, indicating that RIHR had a profound effect on the foregut microflora of laying hens.

#### 3.11.3. Diversity Analysis

At the phylum level, Firmicutes was the dominant group out of all groups, followed by bacteroidota (Figure 8C). At the genus level, the main producing bacteria were *bacteroides*, *unclassified_k__norank_d__Bacteria*, *unclassified_f_Lachnospiraceae* and *Rikenellaceae_RC9_gut_group. Megamonas* abundance was increased in the H-RIHR-H group compared with the H-Control group (Figure 8D).

**Figure 9:**
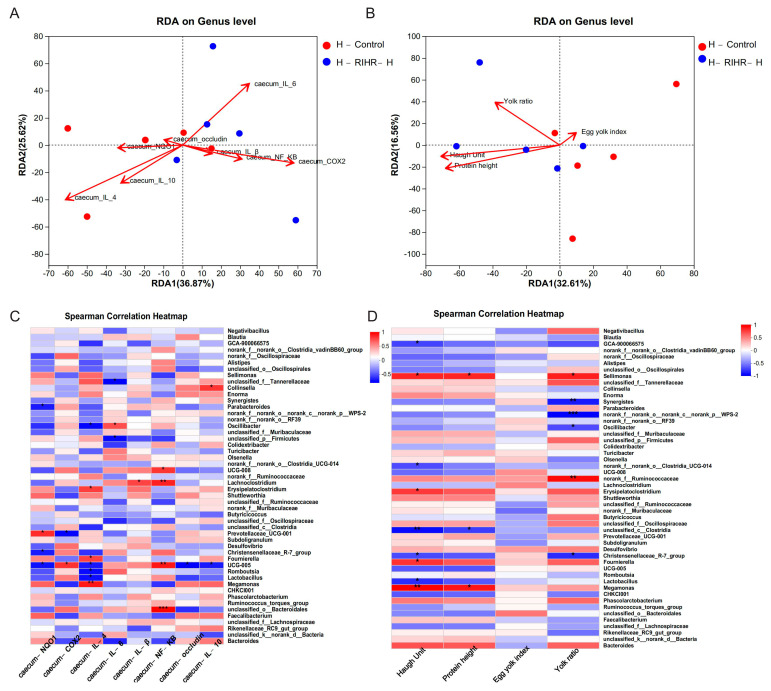
Correlation analysis. (**A**) Analysis of correlation between RDA genus microorganisms and cecum expression genes. (**B**) RDA egg quality correlation analysis. (**C**) Correlation heatmap of cecum expressed genes. (**D**) Heatmap of egg quality correlation. * Indicates significant difference (*p* < 0.05), ** and *** indicate extremely significant differences (*p* < 0.01).

The abundance difference between the two groups at the genus level was analyzed by comparing the species abundance differences in the samples. The results showed that compared with the control group, the abundance level of *Megamonas*, *Sellimonas* was increased (*p* < 0.05) (Figure 8E), and *Faecalibacterium* was increased (*p* > 0.05) (Figure S3). The RDA results showed that compared with H-Control, genus-level microorganisms in the H-RIHR-H group were positively correlated with the anti-inflammatory factors *IL−10* and *IL−4* and negatively correlated with the inflammatory factors *IL−6* and *Cox2* (Figure 9A). On the other hand, RDA also demonstrated the relationship between microorganisms at the hindgut-level and egg-quality-related indicators. Compared with the H-Control group, the posterior intestinal-level microorganisms in the H-RIHR-H group were positively correlated with the Haugh unit and protein height (Figure 9B). The correlation heat map analysis showed that *Lactobacillus* were significantly negatively correlated (*p* < 0.05) with the anti-inflammatory factor *IL−4*, and *Oscillibacter* was significantly negatively correlated (*p* < 0.05) with *IL−4* (Figure 9C). The heatmap of the microbiological correlation between egg quality and the hindgut genus level showed that *Megamonas* was significantly and positively correlated with the Haugh unit (*p* < 0.01). *Sellimonas* was significantly and positively correlated with the Haugh unit, protein height and yolk ratio (*p* < 0.05) (Figure 9D). 

**Figure 10:**
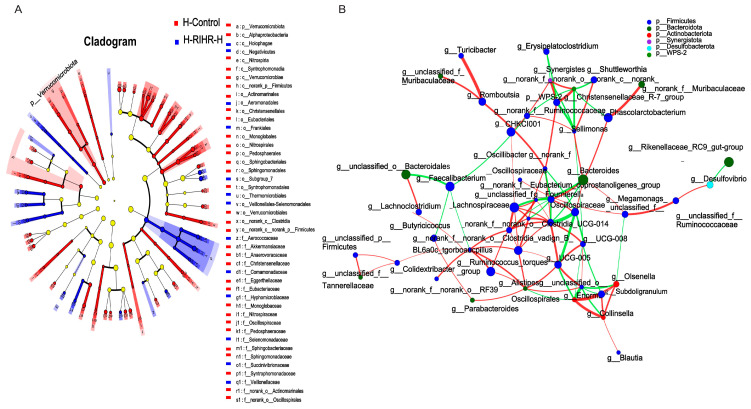
Comparative analysis of intestinal flora after RIHR. (**A**) Lefse analysis. (**B**) Network graph analysis.

The LEfSe analysis also showed that *o_Aeromonadales*, *f_Aerococcaceae* and *f_Veillonellaceae* were significantly enriched in the H-RIHR-H group (Figure 10A). Moreover, the number of positive correlations was significantly greater than the number of negative correlations throughout the network relationships. The probiotic *g_Faecalibacterium* was negatively correlated with other bacteria (Figure 10B).

## 4. Discussion

RIHR, which is derived from the residuals after *R. isatidis* extraction, provides a cost-effective and environmentally sustainable alternative that offers significant economic and environmental benefits, with the dual advantage of reducing production costs while helping to protect the environment [9,19]. More importantly, our study revealed that RIHR holds significant potential in laying hen production, as its active ingredients can improve the eggshell thickness, Ha unit and protein height. Additionally, RIHR has antioxidant and anti-inflammatory effects on the ileum and cecum. Therefore, the ileum and cecum of laying hens that were fed RIHR were quantitatively verified by fluorescence. Interestingly, our study found significant differences in the gene expression between the ileum and cecum. Our results showed that RIHR was able to decrease the levels of inflammatory factors *IL−6* [20] and *TNF−α* [21], while increasing the levels of the anti-inflammatory factor *IL−4* [22] and antioxidant gene *NQO1* [23] in the ileum of laying hens. Similar positive effects were observed in the cecum. However, the levels of inflammatory factors *NF−KB* [24] and *IL−1β* [25] were increased, while the level of the anti-inflammatory factor *IL-10* [26] decreased in the ileum. Additionally, the expression of the intestinal barrier gene *Occludin* [27] decreased in the ileum, but showed the opposite results in the cecum. Therefore, these results indicate that the therapeutic effect of RIHR on intestinal inflammation is more pronounced in the cecum than in the ileum, suggesting a direct action of RIHR on the cecum for treating intestinal inflammation. Regarding antioxidant effects, both the ileum and cecum exhibited positive antioxidant effects, suggesting that RIHR has antioxidant effects on both the ileum and cecum and may improve intestinal health. 

To clarify the active ingredients in RIHR, eight compounds in RIHR were identified using LC-MS, of which three compounds (indirubin, deoxyvasicinone and epigoitrin) are of research value because they have anti-inflammatory and antiviral effects. Previous studies have shown that indirubin has antitumour, antiproliferative and hepatoprotective effects and is also a potent anti-inflammatory agent [28,29]. Deoxyvasicinone is an effective component of medicinal plants such as *Adhatoda Vasica Nees* and *Peganum harmala L*, which exhibit anti-inflammatory, antioxidant, antibacterial and other biological activities with a low toxicity and high safety [30]. Epigoitrin is extracted from the root of *R. isatidis* and has been reported to exhibit antiviral activity by inhibiting viral attachment and proliferation [15]. In addition, Epigoitrin that is extracted from nori, a wild edible herb in northern China, has antioxidant and anti-inflammatory effects and can protect HepG2 cells from H_2_O_2_-induced oxidative damage by enhancing the *Nrf2* response and inhibiting *NF−KB* signaling [31]. The other five compounds, which are not associated with anti-inflammatory and antioxidant effects, are amino acids, indoles and dyes. Consequently, molecular docking predictions were performed for indirubin, deoxyvasicinone and epigoitrin, revealing that indirubin exhibited the strongest binding ability to all docking proteins, followed by deoxyvasicinone and epigoitrin. 

In addition, RIHR can improve the antioxidant performance of laying hens. Previ-ous studies have also indicated that the extract of *R. isatidis* can alleviate oxidative stress [32]. At present, there are few studies on the application of RIHR to the antioxidant effects on laying hens, and most of them focus on the in vitro antioxidant and anti-inflammatory effects of *R. isatidis* extract and its application on host mice [32,33]. Our results indicate that RIHR decreases the MDA content in the liver, increases the SOD content in the liver, and increases the T-AOC level in the yolk. The dietary addition of 15 kg/t RIHR is most effective at increasing the antioxidant capacity of laying hens. In contrast to previous studies of *R. isatidis* extracts, our study lays a scientific foundation for the antioxidant function of RIHR.

In recent years, many studies have proven that intestinal flora plays an important role in maintaining host health, immunity and productivity, and this has become a research focus [34]. In this study, the increased use of RIHR was associated with a decrease in Firmicutes and an increase in Bacteroidetes and Proteobacteria in the foregut. At the genus level, *Lactobacillus* serves as the predominant microflora in the foregut of laying hens and has a strong correlation with feed digestibility [35]. *Lactobacillus* is well known for its multiple roles in maintaining health, helping to protect against pathogens, extract nutrients and energy, and regulate immune function [36,37]. However, our findings show that supplementation with RIHR significantly reduced the levels of *Lactobacillus* in the foregut. In addition, *Lactobacillus* was found to be negatively correlated with other bacteria in the foregut network map, possibly because they produce bacteriocins that inhibit the growth and reproduction of other bacteria [38]. This scenario suggests an intricate interplay among gut microbiota, host immune responses, and external dietary factors like RIHR [39]. One possibility is that RIHR may introduce or enhance the growth of other beneficial microorganisms in the gut, thereby compensating for the decline in *Lactobacillus* and maintaining or even improving the gut’s ecological balance. Furthermore, RIHR itself may contain phytochemicals with anti-inflammatory properties that could mitigate the effects of reduced *Lactobacillus* levels, serving as immunomodulators that suppress the immune response to potential gut pathogens [16,40]. Alternatively, the reduction in *Lactobacillus* could be part of a broader microbial community shift that favors anti-inflammatory species or strains, thereby indirectly reducing intestinal inflammation. This decline could also create an ecological niche that is subsequently filled by bacteria with potent anti-inflammatory or immunoregulatory effects. Another consideration is that the altered microbial composition could modulate metabolic pathways, leading to the production of fewer inflammatory metabolites and a less inflammatory intestinal environment. 

After treating the hindgut with RIHR, there was a notable increase in the levels of *Megamonas*, *Sellimonas* and *Shuttleworthia*. *Megamonas* is typically exclusive to cachexia groups [41], *Sellimonas* [42] is known to be pro-inflammatory, and *Shuttleworthia* is considered potentially pathogenic [43]. It is interesting to observe the elevated levels of beneficial bacteria like *Faecalibacterium* and *Blautia* in the hindgut as well. The network analysis revealed that *Faecalibacterium* is inversely correlated with other bacteria, possibly due to its production of bacteriotin, which inhibits the growth of rival bacteria. In this way, *Faecalibacterium* can improve the intestinal microenvironment and counteract certain pathogens [44]. *Blautia*, which belongs to the phylum Firmicutes and is abundant in mammalian faeces and intestines, shows promise in preventing inflammation and maintaining intestinal homeostasis [45]. However, despite the increase in pathogenic bacteria, there was no corresponding significant increase in beneficial bacteria. Therefore, RIHR may not improve gut inflammation caused by gut microbes, but it acts directly on the intestinal tract of laying hens through the anti-inflammatory compounds of three types of RIHR, inhibiting the expression of *NF−KB*, *COX2*, *IL−1β* and other inflammatory factors, and thereby playing a role in the treatment of inflammation.

## 5. Conclusions

In summary, adding RIHR to the feed of laying hens improves the egg quality, enhances immunity, and increases the antioxidant capacity of ileum and cecum while reducing inflammation. The microorganisms of the foregut microorganisms of the laying hens, *Lactobacillus*, were decreased, and harmful bacteria were increased in the hindgut, while the numbers of the beneficial bacteria did not increase significantly. Therefore, our speculation is that RIHR might not alleviate intestinal inflammation caused by intestinal microorganisms. The mechanism of action could involve the three main active compounds in RIHR acting directly on the intestinal tract of laying hens to inhibit the expression of inflammatory factors like *NF−KB*, *COX2* and *IL−1β*, thus functioning as a therapeutic agent for inflammation. Given the complexity of microbial interactions, recognizing these limitations warrants further research to elucidate the exact mechanisms involved. This exploration could lead to more targeted and effective interventions for poultry health and nutrition. The specific anti-inflammatory mechanisms will be further validated in the future.

## Figures and Tables

**Table 1:** Composition and nutrient levels of experimental basal diet (air-dried basis) %.

Ingredient	Contents (%)	Nutrition Levels	Content (%)
Corn	55	ME/(MJ/kg)	11.48
Soybean	25.1	CP	15.773
Bran	4	EE	2.56
CaHPO_4_	1.5	Lys	0.76
Limestone	9	Met	0.35
Coarse stone grain	2.4	Ca	3.65
^a^ Mineral premix X	3	TP	0.59
Total	100	AP (%)	0.37

^a^ Premix feed provided per kilogram of complete feed: Fe 60 mg, Cu 11 mg, I 0.4 mg, Zn 70 mg, Mn 115 mg, Se 0.30 mg. VA, 6000 IU; VD3, 2500 IU; VE, 25.0 mg; VK3, 2.25 mg; VB1, 1.8 mg; VB2, 7.0 mg; VB6, 4.0 mg; VB12, 0.20 mg; pantothenic acid, 12.0 mg; niacin, 35.0 mg; biotin, 0.14 mg; folic acid, 0.8 mg.

**Table 2:** Names of compounds identified in RIHR (μg/g).

Name of the Compound (μg/g)	Mean ± SEM
L-arginine	1.19 ± 0.19
guanine	1.02 ± 0.39
L-phenylalanine	0.19 ± 0.06
Epigoitrin	0.39 ± 0.05
deoxyvasicinone	0.18 ± 0.05
3-indole acetonitrile	0.52 ± 0.12
indigo	1.90 ± 0.55
indirubin	0.21 ± 0.06

**Table 3:** Effect of RIHR on production performance of laying hens.

Week	Item	Mean ± SEM				*p*-Value
		Control	RIHR-L	RIHR-M	RIHR-H	
first week	Laying rate, %	90.38 ± 2.09	91.13 ± 1.37	89.38 ± 1.83	87.88 ± 1.56	0.586
	Average daily feed intake, g/d	112.36 ± 0.29 ^bc^	108.38 ± 0.42 ^a^	113.01 ± 0.11 ^c^	111.40 ± 0.90 ^b^	0.000
	Feed–egg ratio	1.85 ± 0.01 ^b^	1.76 ± 0.01 ^a^	1.83 ± 0.02 ^b^	1.83 ± 0.01 ^b^	0.001
	Egg weight, g	60.90 ± 0.53	61.55 ± 0.38	61.83 ± 0.61	60.85 ± 0.49	0.450
eighth week	Laying rate, %	91.63 ± 0.73	90.25 ± 1.22	91.50 ± 0.70	89.38 ± 1.81	0.508
	Average daily feed intake, g/d	113.50 ± 0.53	115.83 ± 2.21	113.80 ± 0.88	113.01 ± 0.67	0.425
	Feed–egg ratio	2.01 ± 0.23	2.07 ± 0.07	1.99 ± 0.027	2.05 ± 0.043	0.602
	Egg weight, g	59.43 ± 0.71 ^b^	64.38 ± 1.00 ^a^	64.05 ± 0.83 ^a^	62.65 ± 1.32 ^a^	0.006

Different lowercase letters in the same row indicate significant difference (*p* < 0.05), and the same letters or no letters indicate no significant difference (*p* > 0.05). Low, middle and high doses of RIHR are represented by RIHR-L, RIHR-M and RIHR-H, respectively.

**Table 4:** Effect of RIHR on egg quality of laying hens.

Item	Mean ± SEM				*p*-Value
	Control	RIHR-L	RIHR-M	RIHR-H	
Egg shape index, %	73.24 ± 0.60 ^b^	74.86 ± 0.52 ^ab^	74.76 ± 0.79 ^ab^	76.93 ± 1.06 ^a^	0.020
Eggshell strength, kg ^f^	2.30 ± 0.14	3.32 ± 0.14	3.45 ± 0.07	3.16 ± 0.18	0.120
Yolk color	4.38 ± 0.18 ^a^	4.88 ± 0.13 ^b^	4.88 ± 0.13 ^b^	5.38 ± 0.18 ^c^	0.001
Haugh unit	65.63 ± 2.49 ^b^	74.64 ± 1.99 ^a^	79.68 ± 1.48 ^a^	78.00 ± 1.88 ^a^	0.000
Protein height, mm	6.70 ± 0.26 ^b^	7.83 ± 0.21 ^a^	8.32 ± 0.15 ^a^	8.08 ± 0.20 ^a^	0.000
Egg yolk index	0.34 ± 0.01	0.34 ± 0.01	0.38 ± 0.03	0.38 ± 0.01	0.294
Yolk ratio	0.28 ± 0.00	0.29 ± 0.00	0.30 ± 0.00	0.29 ± 0.01	0.272
Eggshell thickness, mm	0.29 ± 0.01 ^c^	0.37 ± 0.01 ^a^	0.34 ± 0.01 ^ab^	0.33 ± 0.01 ^b^	0.000

Different lowercase letters in the same row indicate significant difference (*p* < 0.05), and the same letters or no letters indicate no significant difference (*p* > 0.05). Low, middle and high doses of RIHR are represented by RIHR-L, RIHR-M and RIHR-H, respectively.

**Table 5:** Effects of RIHR on serum biochemistry of laying hens.

Item	Mean ± SEM				*p*-Value
	Control	RIHR-L	RIHR-M	RIHR-H	
lg M, g/L	0.01 ± 0.00	0.02 ± 0.00	0.06 ± 0.05	0.05 ± 0.03	0.579
lg A, g/L	0.00 ± 0.00	0.00 ± 0.00	0.02 ± 0.02	0.04 ± 0.04	0.524
lg G, g/L	0.01 ± 0.00 ^b^	0.02 ± 0.00 ^a^	0.01 ± 0.00 ^a^	0.01 ± 0.00 ^ab^	0.055
IP, mmol/L	3.02 ± 0.17	2.79 ± 0.20	3.28 ± 0.27	3.30 ± 0.45	0.579
Ca, mmol/L	5.46 ± 0.32	5.35 ± 0.13	5.56 ± 0.25	5.80 ± 0.22	0.606
ALT, U/L	6.86 ± 1.57	8.10 ± 1.89	8.09 ± 1.94	3.50 ± 1.39	0.243
AST, U/L	279.73 ± 16.33	272.61 ± 13.76	251.61 ± 7.44	273.19 ± 10.86	0.431
ALP, U/L	565.04 ± 68.02	769.44 ± 311.20	889.81 ± 355.88	612.03 ± 106.52	0.775
ALB, g/L	25.21 ± 1.35 ^ab^	23.66 ± 0.46 ^b^	25.58 ± 0.98 ^ab^	27.10 ± 1.05 ^a^	0.145
GLU, mmol/L	11.56 ± 0.27	11.34 ± 0.25	11.49 ± 0.20	11.94 ± 0.20	0.319
TG, mmol/L	19.97 ± 2.08 ^ab^	16.24 ± 2.01 ^b^	21.71 ± 1.65 ^ab^	23.74 ± 1.83 ^a^	0.059
TC, mmol/L	4.20 ± 0.58	3.22 ± 0.28	3.71 ± 0.37	4.49 ± 0.44	0.192
HDL-C, mmol/L	1.43 ± 0.10 ^b^	1.42 ± 0.11 ^b^	1.57 ± 0.13 ^ab^	1.92 ± 0.17 ^a^	0.036
LDL-C, mmol/L	0.89 ± 0.08 ^b^	0.83 ± 0.10 ^b^	1.13 ± 0.21 ^ab^	1.45 ± 0.27 ^a^	0.090

Different lowercase letters in the same row indicate significant difference (*p* < 0.05), and the same letters or no letters indicate no significant difference (*p* > 0.05). Low, middle and high doses of RIHR are represented by RIHR-L, RIHR-M and RIHR-H, respectively. Alkaline phosphatase (ALP), aspartate aminotransferase (AST), alanine aminotransferase (ALT), blood phosphorus (IP), calcium (Ca), albumin (ALB), glucose (GLU), total cholesterol (TC), triglyceride (TG), high-density lipoprotein (HDLC), low-density lipoprotein (LDLC), immunoglobulin A (IgA), immunoglobulin G (IgG), immunoglobulin M (IgM).

## Data Availability

Data are contained within the article.

## Supplementary Materials

The following supporting information can be downloaded at https://www.mdpi.com/article/10.3390/antiox12122084/s1: Table S1: Chicken gene-specific primers used for RT-qPCR; Table S2: Contents of conventional amino acids in RIHR; Table S3: Test results of routine indexes in RIHR. Figure S1: Epigoitrin molecular docking; Figure S2: Alpha diversity; Figure S3: Rank sum test for comparison of two groups at the level of hindgut genus. The datasets presented in this study can be found in online repositories. The names of the repository/repositories and accession number(s) can be found below: http://www.ncbi.nlm.nih.gov/ (accessed on 1 September 2023), PRJNA997628.
