# Supplementary material for: The Effects of Radix isatidis Raw Material on Egg Quality, Serum Biochemistry, Gut Morphology and Gut Flora
Source: Antioxidants (Basel). 2023 Dec 7;12(12):2084. doi: 10.3390/antiox12122084 (PMC10741118; doi:10.3390/antiox12122084)
Supplement: Supplementary file 1 [file antioxidants-12-02084-s001.zip › antioxidants-2720873-supplementary.pdf]

Table S1. Chicken gene-specific primers used for RT-qPCR.

**Primer**	**Sequence (5'-3')**	accession number
β-actin	F: GTGATGGCATGGGACATAGCTC<br>R: TGACCATCAGGGAGTTCATAGC	XM_005262365.5
IL-1β	F: TCTGCCTGCAGAAGAAGCC<br>R: CCTCACTTTCTGGCTGGAGG	XM_017001915.2
IL-6	F: CAACCTCAACCTGCCCAA<br>R: GGAGAGCTTCCTCAGGCATT	XM_011537587.4
IL-10	F: CACAACTTCTTCACCTGCGAG<br>R: CATGGCTTTGTAGATCCCGTTC	
		XM_017001211.3
IL-4	F: GTGCCCACGCTGTGC TTAC<br>R: AGGAAACCTCTCCCTGGATGTC	XM_011545394.4
COX2	F: CTGCTCCCTCCCATGTCAGA<br>R: CACGTGAAGAATTCCGGTGTT	XM_047423955.1
NF-κB	F: GATCTGCTGCCCCTGTACCTG<br>R: AGCTGAGCGCÇCTTCACACT	XM_047440102.1
occludin	F: CGGAGCCCAGACTACCAAAG<br>R: TTACACAGCTTCAGCCTTACA	XM_017023084.2
NQO1	F: GTTCAATGCCGTGCTCTCAC<br>R: CCGCTTCAATCTTCTTCTGC	NM_001394034.2
TNF-α	F:TGTGTATGTGCAGCAACCCGTAGT<br>R: GGCATTGCAATTTGGACAGAAGT	XM_047418136.1

Table S2. Contents of conventional amino acids in RIHR.

Nutrient composition	Content (%)
ASP	0.59
GLU	0.80
SER	0.28
HIS	0.22
GLY	0.34
THR	0.30
ARG	0.94
ALA	0.33
TYR	0.15
VAL	0.39
MET	0.05
PHE	0.31
ILE	0.28
LEU	0.46
LYS	0.37
PRO	0.73
CYS	0.07
Total	6.61

Table S3. Test results of routine indexes in RIHR.

Iterm	Mean±SEM
Crude protein, %	12.13±0.00
Energy, cal/g	5147.08±654.45
Moisture, %	6.65±1.89
Coarse ash, %	7.17±0.19
Crude fat, %	7.5±0.78
Coarse fibre, %	8.95±0.06
Ca, %	1.18±0.06
P, %	1.51±0.41

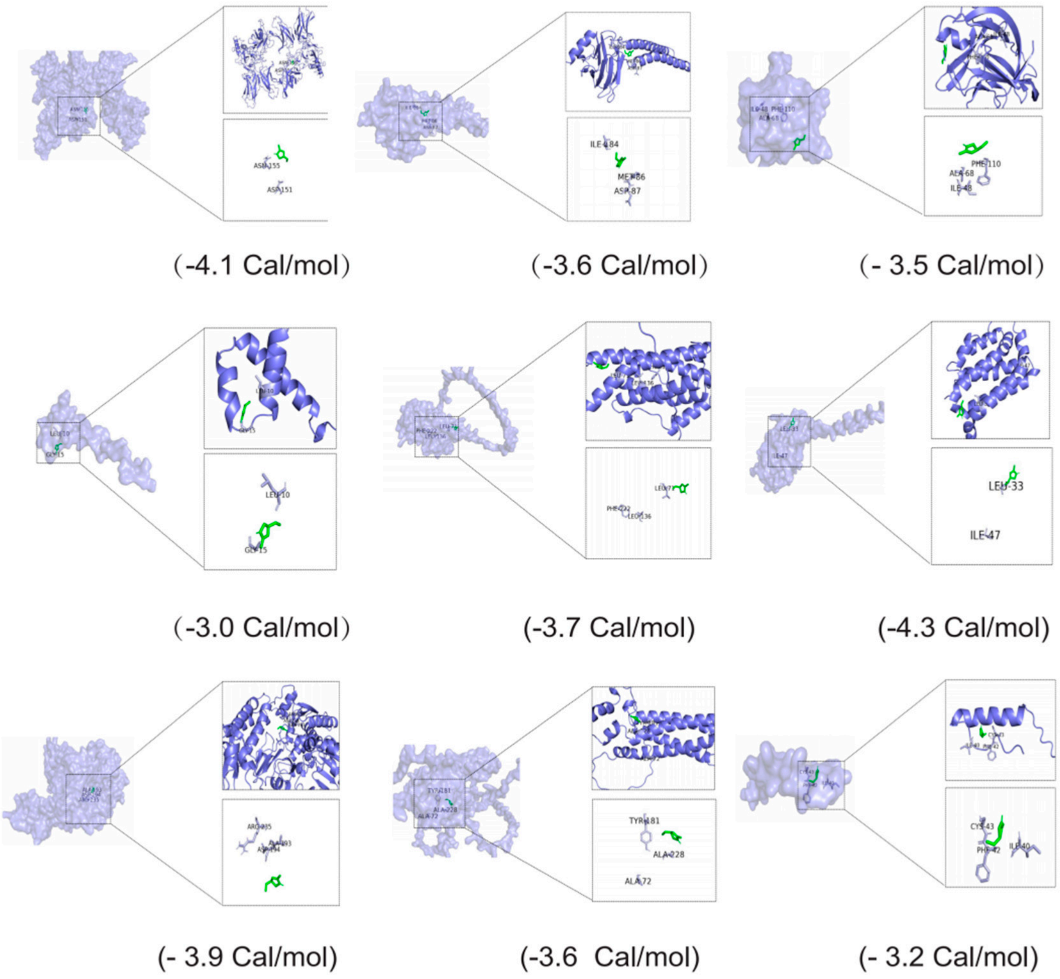

Figure S1. Epigoitrin molecular docking.

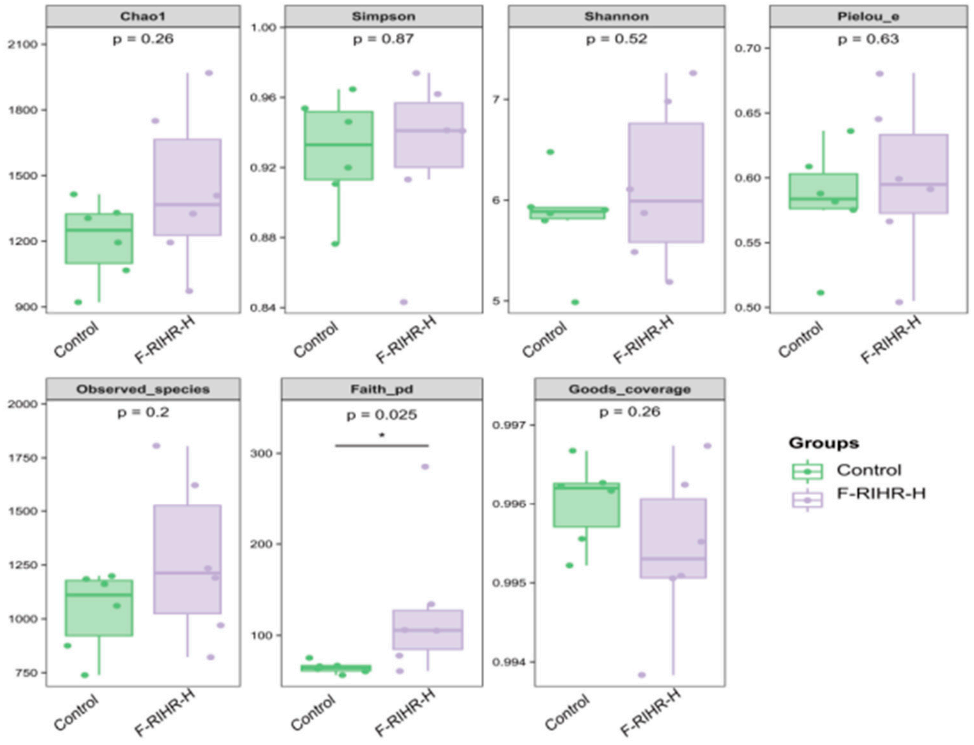

Figure S2. Alpha diversity.

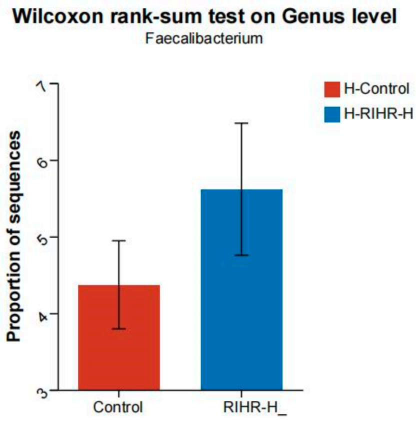

Figure S3. Rank sum test for comparison of two groups at the level of hindgut genus.
